# C-STICH2: emergency cervical cerclage to prevent miscarriage and preterm birth—study protocol for a randomised controlled trial

**DOI:** 10.1186/s13063-021-05464-6

**Published:** 2021-08-11

**Authors:** Victoria Hodgetts-Morton, Catherine A. Hewitt, Laura Jones, Lisa Leighton, Nicole Pilarski, Eleanor Molloy, Kim Hinshaw, Jane Norman, Jason Waugh, Sarah Stock, Jim Thornton, Philip Toozs-Hobson, Tracey Johnston, Arri Coomarasamy, Shakila Thangaratinam, Ben Mol, Eva Pajkrt, Neil Marlow, Tracy Roberts, Lee Middleton, Peter Brocklehurst, Katie Morris

**Affiliations:** 1 University of Birmingham, Birmingham, UK; 2 Birmingham Clinical Trials Unit, Birmingham, UK; 3 Birmingham Women’s and Children’s Hospital, Birmingham, UK; 4 City Hospitals Sunderland NHS Foundation Trust, Sunderland, UK; 5 University of Bristol, Bristol, UK; 6 University of Auckland, Auckland, New Zealand; 7 University of Edinburgh, Edinburgh, UK; 8 University of Nottingham, Nottingham, UK; 9 University of Adelaide, Adelaide, UK; 10 University of Amsterdam, Amsterdamm, UK; 11 University College London, London, UK; 12 Institute of Applied Health Research, University of Birmingham, B15 2TT, Birmingham, UK

**Keywords:** Miscarriage, Preterm birth, Cervical cerclage, Randomised controlled trial, Economic evaluation, Qualitative process evaluation

## Abstract

**Background:**

Cervical cerclage is a recognised treatment to prevent late miscarriage and pre-term birth (PTB). Emergency cervical cerclage (ECC) for cervical dilatation with exposed unruptured membranes is less common and the potential benefits of cerclage are less certain. A randomised control trial is needed to accurately assess the effectiveness of ECC in preventing pregnancy loss compared to an expectant approach.

**Methods:**

C-STICH2 is a multicentre randomised controlled trial in which women presenting with cervical dilatation and unruptured exposed membranes at 16 + 0 to 27 + 6 weeks gestation are randomised to ECC or expectant management. Trial design includes 18 month internal pilot with embedded qualitative process evaluation, minimal data set and a within-trial health economic analysis.

Inclusion criteria are ≥16 years, singleton pregnancy, exposed membranes at the external os, gestation 16 + 0–27 + 6 weeks, and informed consent. Exclusion criteria are contraindication to cerclage, cerclage in situ or previous cerclage in this pregnancy.

Randomisation occurs via an online service in a 1:1 ratio, using a minimisation algorithm to reduce chance imbalances in key prognostic variables (site, gestation and dilatation). Primary outcome is pregnancy loss; a composite including miscarriage, termination of pregnancy and perinatal mortality defined as stillbirth and neonatal death in the first week of life. Secondary outcomes include all core outcomes for PTB. Two-year development outcomes will be assessed using general health and Parent Report of Children’s Abilities-Revised (PARCA-R) questionnaires. Intended sample size is 260 participants (130 each arm) based on 60% rate of pregnancy loss in the expectant management arm and 40% in the ECC arm, with 90% power and alpha 0.05. Analysis will be by intention-to-treat.

**Discussion:**

To date there has been one small trial of ECC in 23 participants which included twin and singleton pregnancies. This small trial along with the largest observational study (*n* = 161) found ECC to prolong pregnancy duration and reduce deliveries before 34 weeks gestation. It is important to generate high quality evidence on the effectiveness of ECC in preventing pregnancy loss, and improve understanding of the prevalence of the condition and frequency of complications associated with ECC. An adequately powered RCT will provide the highest quality evidence regarding optimum care for these women and their babies.

**Trial registration:**

ISRCTN Registry ISRCTN12981869. Registered on 13th June 2018.

## Background

Prevention of second trimester miscarriage and preterm birth (PTB) are important public health priorities. Stillbirth and delivery before 37 completed weeks of gestation are major contributors to pregnancy loss and the prevalence of significant morbidities among survivors following PTB at low gestational ages remains of concern, despite improving survival (BAPM Framework ADC 2019). These two conditions frequently share common aetiologies, namely cervical insufficiency and infection [14] and the management of women at risk of miscarriage or PTB remains broadly similar. Cervical cerclage by placing a stitch around the cervix has been shown to be effective at reducing the risk of second trimester miscarriage or PTB in women with a closed cervix and cervical insufficiency identified by clinical history or ultrasound findings [13]. Planned cerclage or cerclage in response to a shortened cervix is associated with an increased proportion of deliveries above 35 weeks [2].

Less frequently, in the second trimester a woman may present with a cervix that is already dilated exposing the foetal membranes. In this situation an emergency cervical cerclage (ECC) or ‘rescue’ stitch can be attempted, with the intention of prolonging the pregnancy, preventing miscarriage or very early PTB, with the goal of improved neonatal outcomes [1]. The risks of this procedure are poorly described and are likely to be higher than in a planned procedure with a closed cervix [19]. These include a risk of membrane rupture during the procedure, leading to pregnancy loss or delivery at an earlier gestation than would have occurred without intervention [6]. In addition, there is risk of infection and potential harm to the mother or her baby, a risk of damage to the cervix and a risk of bleeding [10].

Few studies have investigated ECC. The majority have used an observational design [10], with one randomised controlled trial (RCT). This RCT included only 23 women, 13 allocated to ECC plus indomethacin and 10 to bed-rest [1]. The trial could not separate out the effect of indomethacin as a tocolytic from cerclage. Furthermore, over one third of the participants in this trial had twin pregnancies; in contrast to singleton pregnancies, there is no evidence to support the use of cerclage in multiple pregnancy [15]. Despite the small trial size and these limitations, the mean time to delivery was longer in the ECC arm (54 vs 20 days, *p* = 0.05) and the proportion who delivered before 34 weeks was reduced (54% vs 100%, *p* = 0.02) [1].

The evidence for ECC was reviewed by NICE [11], which concluded that there may be benefit from ECC but that further evidence was required. In response to this we have designed C-STICH2 (Emergency Cervical Cerclage to Prevent Miscarriage and Preterm Birth), an open, multicentre randomised controlled trial to determine whether ECC improves maternal and infant outcomes in women who present with cervical dilatation and exposed unruptured foetal membranes, compared to routine expectant management. This protocol describes an internal pilot to determine feasibility and the procedure for a full RCT.

## Methods/design

### Aims and objectives

The aims and objectives of the C-STICH2 trial are presented in Table 1.

**Table 1:** Description of the aims and objectives of C-STICH2 internal pilot and full trial

Aim: To evaluate whether ECC can improve outcomes for mothers and babies’ in women who present with cervical dilatation and exposed unruptured foetal membranes.	
**Pilot objectives**	• To ascertain if the trial and trial processes are acceptable to women, including the ability to recruit and randomise women. • To assess whether the event rate of the primary outcome is compatible with the estimate used in the sample size calculation. • To explore if clinicians are in equipoise and willing to randomise to an RCT.
**Full trial objectives**	• To determine whether an ECC reduces pregnancy loss (miscarriage, termination of pregnancy, stillbirth or neonatal death within 7 days of delivery) in women who present with cervical dilatation and exposed, unruptured foetal membranes between 16 + 0 and 27 + 6 weeks. • To follow up all surviving babies to 2 years of corrected age to determine their general health and medium-term neurodevelopmental outcomes. • To determine the complication rates of ECC: iatrogenic rupture of membranes during the procedure; insertion failure; predictors of successful ECC placement such as magnitude of dilatation

### Study design and setting

C-STICH2 is an open, multicentre, superiority, RCT in the UK. There is an internal pilot with an embedded qualitative process evaluation. The full trial includes a within-trial economic analysis and a minimal dataset of anonymised demographic and pregnancy outcome data from all women presenting with the condition who do not consent to participate in the randomised arms of C-STICH2.

### Participants

Women will be considered eligible for C-STICH2 if they are aged 16 years or over, with a singleton pregnancy and in whom the cervix is found to be dilated with the foetal membranes intact and exposed, at or below the level of the external os, based on judgement. This may be diagnosed on speculum examination or by ultrasound finding, if performed by a suitably trained practitioner. The gestational age of the pregnancy must be 16 + 0 to 27 + 6 weeks based on the best available estimate and the woman must be able to provide written informed consent. The exclusion criteria are as follows: contra-indication to ECC as judged by the responsible clinician; if the participant has already received cervical cerclage of any type in this pregnancy, or has a cerclage in situ from a previous pregnancy.

### Participant enrolment

**Fig. 1:**
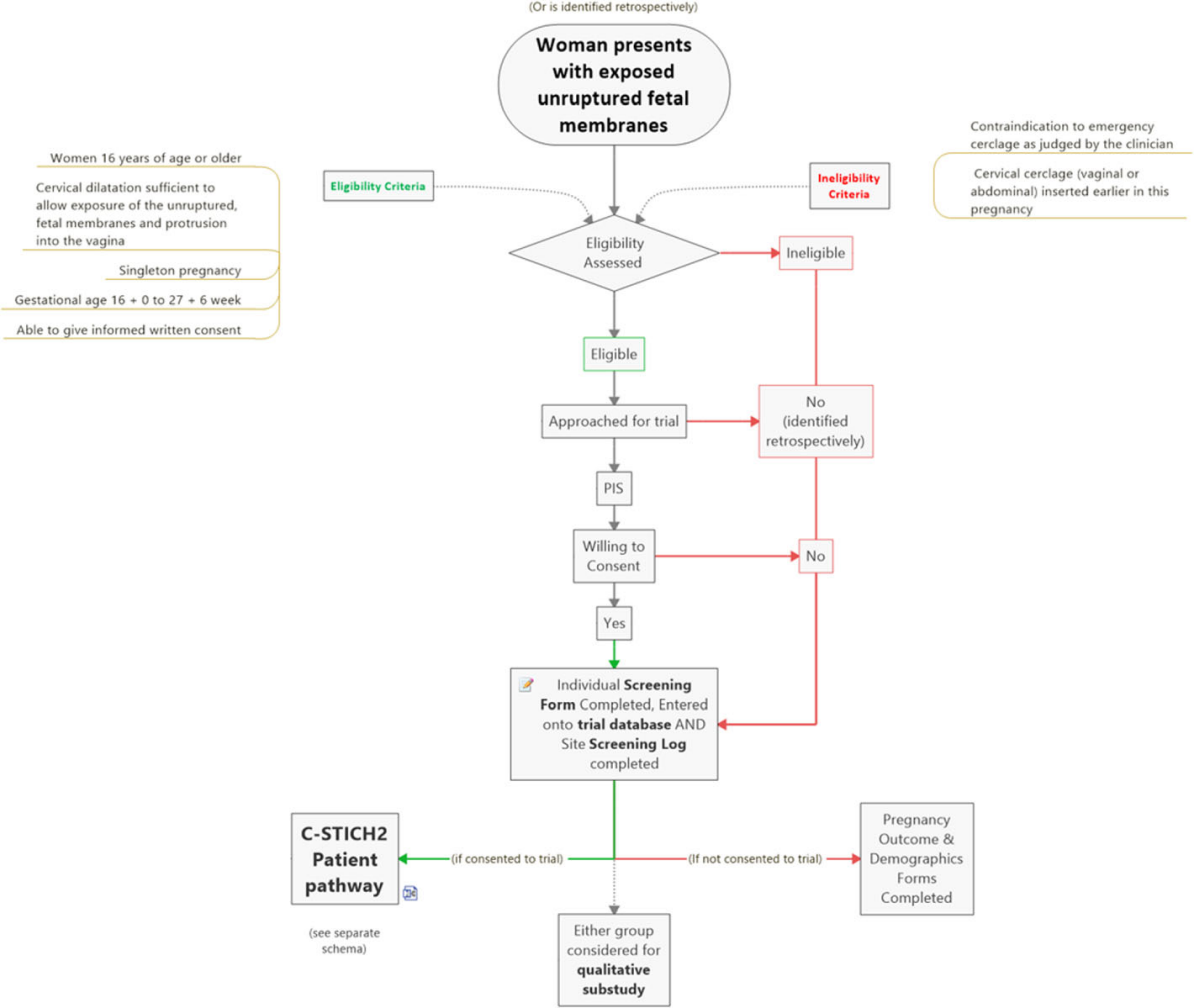
C-STICH2 flow diagram

Participants will be identified following clinical presentation to maternity assessment units, or women may be identified through screening in a specialist PTB clinic setting (Fig. 1). Women who meet the eligibility criteria should be approached by the most senior clinician available about participating in the trial. It is recognised that this may be a very difficult time for women and their families and sufficient time will be given for women to consider participation and explore options. The clinician should be familiar with counselling women at risk of preterm delivery and have received training from the trial team or principal investigator. A detailed participant information sheet (PIS) will be provided. If the woman agrees to participate, written consent must be obtained prior to randomisation. Participants must understand that they are free to withdraw from the trial at any time and that this will not affect their subsequent care.

In women who decline to participate in the randomised arms of C-STICH2 or who are not approached regarding the study, a non-consented anonymised minimal dataset will be collected. This dataset includes basic demographics and pregnancy outcome data to inform overall prevalence of the condition and incidence of pregnancy loss.

### Qualitative process evaluation

All women approached about the trial should be informed about the qualitative process evaluation, even if they have chosen to decline trial participation. They will receive written information about participation in the qualitative study either at the time of presentation and trial approach, shortly after at a time considered appropriate by their healthcare professionals (HCPs), at a follow-up appointment, or via a follow-up letter. Women who express an interest in participating will, where feasible, be asked to complete and sign a consent to contact form giving written permission to be contacted by the qualitative research team. Women who are sent a follow-up letter about the qualitative study will be asked to return a completed consent to contact form or will be able to contact the research team directly. Following receipt of the consent to contact form or direct contact, the qualitative team will assess suitability of contact timing with the recruiting site. If this is deemed to be appropriate, the team will then discuss participation with the woman and separate informed consent (written, electronically completed or verbal) for the qualitative study will be obtained. The qualitative process evaluation will use semi-structured interviews to explore women’s experiences of diagnosis and care, and their views of the trial processes irrespective of their decision to participate in C-STICH2.

HCPs named on the site delegation logs will also be approached directly by the qualitative research team to participate in semi-structured interviews to help inform our understanding of the following: issues approaching women to participate, views on barriers and facilitators to trial recruitment, thoughts and experiences on the use of ECC including personal and community equipoise. All participants will complete a demographic questionnaire to inform sampling and subsequent description of participant characteristics.

Women and HCPs will be able to choose how they participate in an interview (e.g. face to face, via phone, or via video calling). Interviews will be digitally-audio recorded with data collection and initial analysis taking place iteratively [3]. We anticipate undertaking up to 50 interviews in total but the numbers will remain flexible to ensure that we collect sufficiently rich data to address the aims and objectives of the study [8]. Audio files will be transcribed clean verbatim by a specialist transcription company and the framework approach [4] used to facilitate a systematic and flexible approach to the analysis.

The findings from the qualitative process evaluation will dynamically inform trial design both during and subsequent to the pilot [12]. The embedded qualitative findings will influence trial processes moving beyond the pilot study, inform trial steering committee (TSC) recommendations and help to maximise recruitment and retention in this challenging setting.

### Randomisation and blinding

After eligibility has been confirmed and informed consent obtained, women can be randomised into the C-STICH2 trial (Fig. 1). Randomisation will be provided by a secure 24/7 online randomisation system and a backup free telephone randomisation service (available during working hours). Randomisation can be completed by any staff member on the delegation log assigned to do so. Participants will be randomised in a 1:1 ratio to either ECC or expectant management. A minimisation algorithm will be used to ensure balance in the treatment allocation by site, gestation (16 + 0–19+ 6/20+ 0–23+ 6/24+ 0–27+ 6 weeks) and cervical dilatation (≤3 cm ≥ 4 cm/fully dilated-minimal cervix felt). A ‘random element’ will be included in the algorithm, so that each participant has a probability (unspecified here) of being randomised to the opposite treatment that they would have otherwise received.

It is not possible to blind women or clinicians to the intervention they have received as the comparison is between a surgical intervention and expectant management. The primary outcome (miscarriage, stillbirth, termination of pregnancy and neonatal death) is, however, objective and thus should minimise bias.

### Trial interventions

This trial will be pragmatic in nature allowing the use of treatment adjuncts such as antibiotics or indomethacin in both the ECC and expectant management arms following randomisation.

For women allocated to ECC, this should be delivered at a time that is felt to be clinically appropriate and within 72 h of randomisation. An ECC will involve, under appropriate anaesthesia, the replacement of the foetal membranes into the uterine cavity and the placement of a “purse string” cervical cerclage around the body of the cervix, aiming to occlude the cervical canal and prevent further prolapse of the foetal membranes. Pre-operative, operative, and post-operative management is at the discretion of the clinician responsible for the woman.

Women allocated to expectant management will be managed pragmatically according to local protocols. If an ECC is placed, this will be considered a protocol deviation.

### Follow-up

Surviving children will be followed up at 2 years of corrected age. On discharge from hospital, consent for long-term follow-up will be confirmed and contact details for the main carer will be collected. The research team will maintain contact with families of surviving children at intervals to minimise loss to follow-up. At 2 years corrected age, the main caregiver will be sent questionnaires, this will include general health, Parent Report of Children’s Abilities-Revised (PARCA-R) questionnaire [7, 9] and an assessment of motor outcomes.

### Adverse event reporting

Adverse outcomes, both maternal and neonatal, are common in this high-risk obstetric population. Adverse events (AEs) will be reported via case report forms (CRF) and captured via pre-defined outcome measures. Serious adverse events (SAEs) including but not limited to maternal admission requiring care in high dependency units (HDU) or intensive treatment units (ITU), life-threatening maternal conditions and maternal complications associated with ECC will be reported directly to the trial office and may require expedited reporting. It is recognised that some SAEs are to be expected in these women either as an outcome or known risk of the intervention and whilst it is important that they are reported, this can be done within the relevant CRF. Examples include but are not limited to miscarriage, preterm delivery, admission to hospital for delivery or removal of suture, damage to cervix during ECC insertion or admission to a neonatal unit.

### Outcome measures

The primary outcome is pregnancy loss defined as miscarriage, termination of pregnancy and perinatal mortality defined as stillbirth and neonatal death in the first week after birth. Secondary outcomes are detailed in Table 2. All the core outcomes for PTB are included [17].

**Table 2:** Full description of all secondary outcomes including complete core outcome set for preterm birth

**Secondary outcomes—maternal, neonatal and paediatric**	
**Maternal**	
Pregnancy loss (miscarriage, termination of pregnancy and perinatal mortality, including any stillbirth or neonatal death in the first week of life). Excluding those due to congenital anomalies (chromosomal and/or structural) assessed via death certification.	
Time from conception to pregnancy end (any reason)	
Miscarriage and pre-viable neonatal death (defined as delivery < 24 weeks)	
Stillbirth (defined as intrauterine death ≥ 24 weeks)	
Gestation at delivery	
Pre-term delivery (pre-specified groups of ≤ 28/≤ 32/≤ 37 weeks)	
Maternal sepsis (at any time in pregnancy and until discharge from hospital postnatally)	
Preterm (< 37 weeks) pre labour rupture of membranes (> 24 h prior to delivery) (PPROM) adjusting for gestational age at occurrence of membrane rupture	
Mode of initiation of birth (spontaneous or iatrogenic)	
Indication for iatrogenic delivery (maternal and/or foetal)	
Mode of delivery (vaginal or operative vaginal or caesarean)	
Cerclage placement complications assessed as a composite and individually: cervical laceration; bleeding from cervix; ruptured membranes; bladder injury	
Cerclage removal complications assessed as a composite and individually: cervical tears; difficulty in removal defined as requiring unexpected anaesthesia or unexpected dissection of suture	
Suspected or confirmed chorioamnionitis (during pregnancy and up to 7 days postnatally)	
Maternal admission to HDU or ITU pre-delivery	
Maternal admission to HDU or ITU post-delivery	
Serious adverse events	
**Neonatal**	
Early neonatal death (defined as a death within 7 days after delivery)	
Late neonatal death (defined as a death beyond 7 days and before 28 days after delivery)	
Early neonatal death (defined as a death within 7 days after delivery excluding those secondary to congenital anomalies)	
Late neonatal death (defined as a death beyond 7 days and before 28 days after delivery excluding those secondary to congenital anomalies)	
Birth weight adjusted for gestational age and sex	
Small for gestational age (< 10th centile)	
Advanced resuscitation at birth (assisted ventilation and/or drug administration and/or cardiac compressions)	
Admission to specialist care (SCBU/NICU/HDU/transitional care)	
Length of stay in each additional specialist care setting	
Suspected sepsis (clinically diagnosed defined as commenced on intravenous antibiotics for > 48 h after birth)	
Confirmed sepsis (positive microbiology)	
Brain injury (defined as any intraventricular haemorrhage (IVH) (excludes subependymal haemorrhages), parenchymal cystic or haemorrhagic lesion or persistent ventriculomegaly (VI > 97th percentile)	
Respiratory support (ventilation/CPAP)	
Days on respiratory support	
Supplementary oxygen requirements at 36 weeks corrected gestational age	
Necrotising enterocolitis (Bell’s stage 2 or 3)	
Retinopathy of prematurity requiring laser treatment	
Disabilities	
Congenital abnormalities	
Serious adverse events	
**Paediatric outcomes**	
Death at greater than 28 days until 2 years	
At 2 years corrected age, outcomes obtained from questionnaires, which includes general health, PARCA-R questionnaire and an assessment of motor outcomes (further details given in the statistical analysis plan).	

### Sample size estimates

**Fig. 2:**
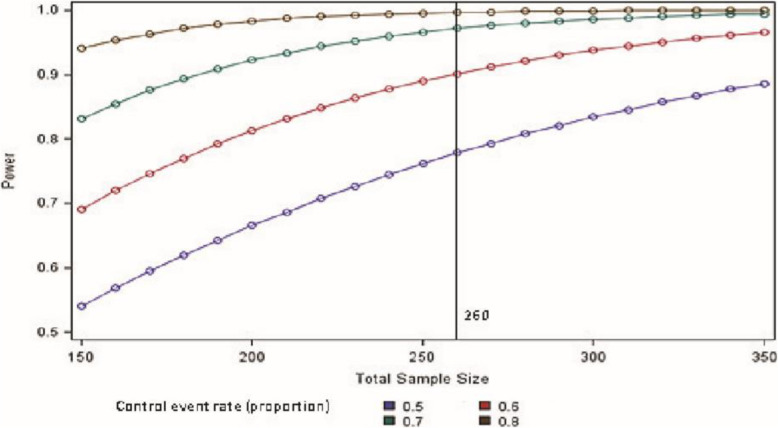
Power curves assuming 33% relative risk reduction and event rates of 50%, 60%, 70% and 80% in the expectant management arm

Published literature [1, 2] noted large effect sizes in favour of cervical cerclage. We accept these effect sizes are likely to be exaggerated due to methodological limitations of these studies and so have opted for a more conservative target difference of a 33% relative risk reduction from 60% pregnancy loss in the expectant management group to 40% in the ECC group. To detect this difference with 90% power (*α* = 0.05) we require 260 women in total (130 in each arm). Smaller differences are also likely to be clinically relevant. The pregnancy loss rate in the expectant management arm is uncertain; however, 260 women would still achieve high levels of power (≥ 80%) in scenarios where the event rate approaches high levels (Fig. 2).

### Statistical analysis

A separate statistical analysis plan for the quantitative analysis of the C-STICH2 study will provide a detailed description of the planned statistical analyses. A brief outline is given below.

Pregnancy loss will be summarised by treatment arm using frequencies and percentages. A log-binomial model will be used to generate relative risks (and 95% confidence intervals (CI)), adjusting for the minimisation variables.

Secondary outcomes which are binary will be analysed as per the primary outcome. Continuous outcomes which are deemed to be normally distributed will be summarised using means and standard deviations and a linear model will be fitted to generate adjusted mean differences (and 95% CIs). Continuous outcomes which are not deemed to be normally distributed will be summarised using medians and interquartile ranges and unadjusted differences in medians will be produced with 95% CIs. Time to event data will be summarised using medians and interquartile ranges. A Cox regression model will be fitted to generate adjusted hazard ratios (and 95% CIs) and a Kaplan-Meier plot will be produced to assess the data visually. All analyses will be adjusted for the minimisation variables (where possible).

All analyses will be by intention to treat. Every attempt will be made to collect pregnancy outcome data on all women, and it is anticipated that missing data will be minimal. Women with missing primary outcome data will not be included in the first instance. Sensitivity analyses will be conducted to assess the impact of missing data. Subgroup analyses will be limited to gestational age at randomisation, cervical dilatation and use of adjuvant therapies.

### Health economics

The principal economic analysis will be based on the data collected within the trial and will relate only to the initial period of assessment and the principal outcome of the trial at 7 days. The results of the economic evaluation will be expressed in terms of major outcomes averted (MOA), where MOA represents a composite outcome based on miscarriage, termination of pregnancy, neonatal death and PTB.

A further analysis based on all data up to the infant reaching 2 years of corrected age will be carried out; the outcome of this second analysis will be based on the parent report and neurodevelopment at 2 years.

The economic evaluation will primarily take the perspective of the NHS and Personal Social Services (PSS) and as far as possible, depending on available data in the literature, will also be analysed from the societal perspective. Data will be collected prospectively on NHS resource from all participating centres for both arms of the trial and follow-up care. Unit costs from routine sources will be attached to resource use to estimate overall costs for each trial arm.

A bootstrapping approach will be used to calculate confidence intervals around the difference in mean costs. Initially, the base-case analysis for the within trial analysis will be framed in terms of cost-consequences, reporting data in a disaggregated manner on the incremental cost and the important consequences as assessed in the trial arms. An incremental economic analysis will be conducted on the primary outcome and other secondary outcomes. The results of these economic analyses will be presented using cost-effectiveness acceptability curves to reflect sampling variation and uncertainties in the appropriate threshold cost-effectiveness value. Simple and stochastic cost-effectiveness analyses will explore the robustness of the results to plausible variations in key assumptions and variations in the analytical methods used, and to consider the broader issue of the generalisability of the results.

### Trial management and oversight

The trial is registered with the ISRCTN registry - trial reference ISRCTN12981869.

The trial is funded by NIHR HTA (project number 16/151/01). The sponsor is Birmingham Women’s and Children’s NHS Foundation Trust. Neither the funder nor sponsor is involved in data collection or analysis.

The trial will be administered by a clinical trials unit with extensive experience. Data will be kept in accordance with General Data Protection Regulations 2018. Completed CRFs will be reviewed by the clinical trials unit and missing or ambiguous data queried. The sponsor will ensure data integrity through quality assurance processes and audit at participating sites.

Any changes to the protocol will be agreed by the TSC prior to implementation and these will be disseminated to individual sites by the trial management group (TMG) subject to research ethics committee approval.

The TMG are responsible for the day to day running of the trial. The TSC and data monitoring committee (DMC) provide independent oversight of the trial including an assessment of the pilot study at the end of year two in line with the pre-specified objectives. TSC members include a majority of members who are independent of the investigators, their employers, institutions and the funding body. The DMC comprises three independent members (two obstetricians and a statistician with extensive trial experience) who are responsible for reviewing interim analyses. Responsibility for continuation or modification of the trial is held by the TSC and will include guidance from the DMC. The terms of reference and charter for this DMC will be guided by the DAMOCLES project, and we anticipate the DMC and TSC will meet biannually.

### Pilot evaluation

The internal pilot comprises the first 18 months of recruitment to C-STICH2. Following this, the feasibility of the trial will be assessed on its ability to screen, randomise and follow-up women. To support the quantitative assessment of feasibility a qualitative process evaluation is fully embedded in this pilot phase and will explore the feasibility, acceptability and appropriateness of the trial and intervention for HCPs and women (as described previously).

We have limited the use of fixed stop/go criteria for the pilot aiming to evaluate the strengths and weaknesses of the proposed trial qualitatively proposing solutions to the challenges experienced. This is partly because so little is known about the prevalence of the condition and its sequelae. Further information will be obtained from the minimal dataset.

**Fig. 3:**
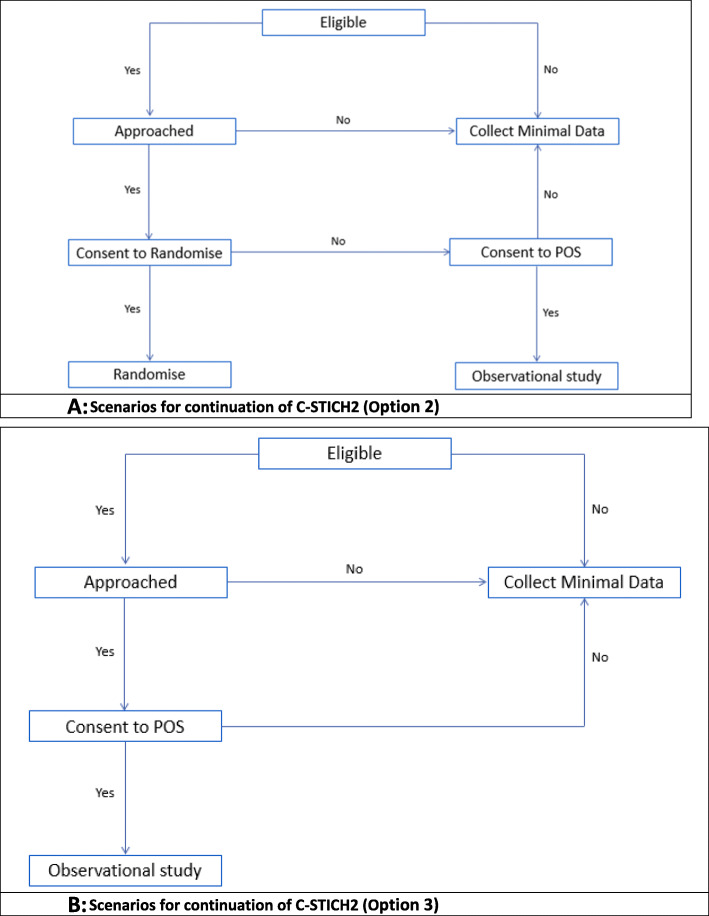
Scenarios for continuation of C-STICH2 if amendments required post pilot evaluation. **A** Scenarios for continuation of C-STICH2 (Option 2). **B** Scenarios for continuation of C-STICH2 (Option 3)

Following the pilot phase of the trial the TMG and oversight committees will review the study progress and make recommendations to the funder on how the project should proceed. We anticipate multiple possible scenarios for how the project could proceed following the internal pilot, whilst maintaining the central research question. These are as follows:

1. The RCT is deemed feasible and remains unchanged (as detailed previously).
2. The RCT is deemed semi-feasible, and a change is required. Recruitment is considered feasible; however, recruitment rates are not as anticipated and it is considered unlikely we will reach the original sample size target of 260 women within the propose timelines. Thus, the RCT could be potentially underpowered within the original sample size parameters. Alternative analysis methods will be considered. In this scenario, a prospective observational cohort study (POS) would be implemented to run in parallel to the trial, aiming to collect detailed information from eligible women who have declined participation in the randomised cohort or where the randomised trial was not offered to the women (Fig. 3A). Ineligible women or those who do not consent to the POS will be considered for the minimal dataset only (as per methods described previously). The POS data will aim to support findings from the RCT.
3. The RCT is considered not feasible and is discontinued. In this scenario, the project would proceed with a POS and minimal dataset only (Fig. 3B).

## Discussion

C-STICH2 is an RCT assessing the effectiveness of ECC in preventing pregnancy loss in mothers with cervical dilatation and exposed, unruptured membranes between 16 + 0 and 27 + 6 weeks of gestation. The situations where ECC may be considered are uncommon, including the absence of significant infection, heavy bleeding or active labour [18].

We anticipate that this trial will be challenging to run but, as indicated by NICE, it is important to provide evidence on which to base management decisions where ECC is potentially indicated. The presence of cervical dilatation and exposed membranes is frequently unanticipated by the woman and clinicians as symptoms may be mild without predisposing factors. Intervention with ECC is time critical in a situation where discussions about prognosis and survival of the woman’s foetus are needed. Recruitment to an ongoing RCT at such a time is distressing and adds further uncertainty for the woman and her family. Furthermore not all senior clinicians are competent or comfortable performing this procedure.

Thus there is a need for a robust RCT of ECC, with an RCT being the gold standard to determine the effectiveness of an intervention [5]. Observational data cannot demonstrate causality and are subject to confounding factors and bias [16]. The observational data already available are limited and at risk of bias [10]. The only completed RCT to date was too small and limited to inform practice [1].

C-STICH2 was designed to pro-actively address as many of these challenges as possible. The decision to leave eligibility primarily to the discretion of the senior clinician, as well as, peri-operative decision making and the use of any treatment adjuncts ensures the trial findings reflects clinical practice in this complex scenario. In addition, the trial facilitates professionals to work within the limits of their own equipoise in relation to the use of ECC. The internal pilot includes an embedded qualitative process evaluation and has clear objectives and scenarios for continuation. This will allow the TSC to consider the trial design and make amendments where appropriate to address any issues identified within the pilot. The minimal dataset will provide information on the prevalence of the condition and important outcomes which is important in understanding the natural history in women who are not eligible or decline randomisation. Each stage of the trial has been designed to be as pragmatic as possible, in order to maximise acceptability of the trial for clinicians and meet the needs of women participating.

In a trial of surgical intervention versus no intervention, it is not often possible to blind participants or HCPs to the intervention received. Whilst an open-label trial can potentially introduce bias, many of the outcomes and in particular the primary outcome (pregnancy loss) are objective and hence subject to less bias.

**Fig. 4:**
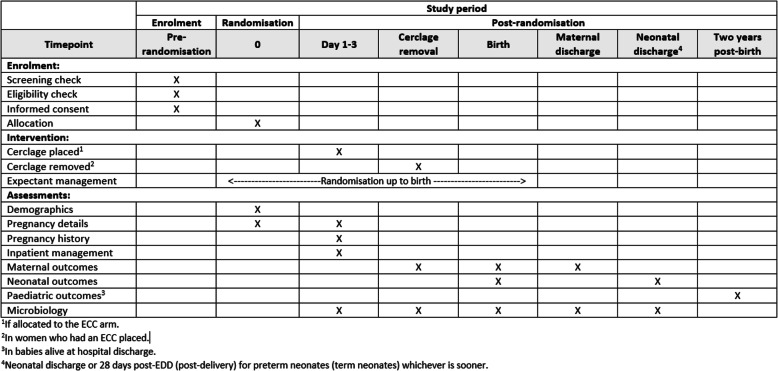
SPIRIT figure

The qualitative process evaluation is key to this trial and will facilitate learning from the experiences of women and HCPs such that improvements can be made to improve care provision for these women. Findings of the qualitative study will allow the trial team to address barriers to participation where identified, build on facilitators, promote examples of good practice and explore concerns of both participants and HCPs. These will feedback into the design and conduct of the trial moving forward through the pilot period and into the full trial (Fig. 4).

## Patient and public involvement

Patients and patient groups are key to this programme being successful. They have been involved in developing the C-STICH2 trial protocol which included informing the choice of primary outcome and advising on the acceptability of trial processes. We have one patient co-applicant and two service user representatives. Our patient representative has direct experience of cervical cerclage and supports others through cerclage and second trimester pregnancy loss. Our two service user representatives, one from Tommy’s and one from the Miscarriage Association, have extensive experience of working with women and their families who have experienced miscarriage and/or PTB and so are ideally placed to support C-STICH2. Our patient representative sits on the TMG and our Miscarriage Association and Tommy’s representatives sit on the TSC providing important stakeholder oversight into trial management and decision making. Throughout the duration of the trial, our patient representative will continue to provide important insight into participant perspectives; support the development of all participant facing materials; contribute to data collection, analysis and interpretation of the qualitative process evaluation data; help to identify and overcome trial issues, and ensure appropriate dissemination of the findings of the study to women, their families and policymakers.

## Trial status

C-STICH2 opened to recruitment on the 1st February 2019. The pilot study is due to complete in August 2020 following which the TSC will make their recommendation. The full trial is expected to recruit for a further 2 years, with 2 years for follow-up therefore completing August 2024. Upon completion of the trial, findings will be disseminated to participants and published in a peer-reviewed journal.

This article is based on the current protocol V3.0; 31.01.2020.

## Data Availability

Requests for data generated during this study will be considered by BCTU. Data will typically be available within six months after the primary publication. Only scientifically sound proposals from appropriately qualified Research Groups will be considered for data sharing. The request will be reviewed by the BCTU Data Sharing Committee in discussion with the Chief Investigator and, where appropriate (or in absence of the Chief Investigator) any of the following: the Trial Sponsor, the relevant Trial Management Group (TMG) and independent Trial Steering Committee (TSC). A formal Data Sharing Agreement (DSA) may be required between respective organisations once the release of the data is approved and before data can be released. Data will be fully de-identified (anonymised) unless the DSA covers the transfer of patient identifiable information. Any data transfer will use a secure and encrypted method.

## References

[1] Althuisius SM, Dekker GA, Hummel P, van Geijn HP (2003). Cervical incompetence prevention randomized cerclage trial: emergency cerclage with bed rest versus bed rest alone. Am J Obstet Gynecol.

[2] Berghella V, Rafael TJ, Szychowski JM, Rust OA, Owen J (2011). Cerclage for short cervix on ultrasonography in women with singleton gestations and previous preterm birth: a meta-analysis. Obstet Gynecol.

[3] Dicciccio-Bloom B, Crabtree BF (2006). The qualitative research interview. Med Educ.

[4] Gale NK, Heath G, Cameron E, Rashid S, Redwood S (2013). Using the framework method for the analysis of qualitative data in multi-disciplinary Health Research. BMC Med Res Methodol.

[5] Hariton E, Locascio JJ (2018). Randomised controlled trials - the gold standard for effectiveness research. BJOG Int J Obstet Gynaecol.

[6] Ito A, Maseki Y, Ikeda S, Tezuka A, Kuribayashi M, Furuhashi M (2017). Factors associated with delivery at or after 28 weeks gestation in women with bulging fetal membranes before 26 weeks gestation. J Matern Fetal Neonatal Med.

[7] Johnson S, Wolke D, Marlow N (2008). Developmental assessment of preterm infants at 2 years: validity of parent reports. Dev Med Child Neurol.

[8] Malterud K, Siersma VD, Guassora AD (2015). Sample size in qualitative interview studies: guided by information power. Qual Health Res.

[9] Martin AJ, Darlow BA, Salt A, Sebastian L, McNeill N, Tarnow-Mordi W (2013). Performance of the parent report of Children's abilities-revised (PARCA-R) versus the Bayley scales of infant development III. Arch Dis Childhood.

[10] Namouz S, Poraz S, Okun N, Windrim R, Farine D (2013). Emergency cerclage: literature review. Obstetric Gynecol Survey.

[11] National Institute for Clinical Excellence (NICE). Preterm labour and birth guideline 25. 2015.

[12] O'Cathain A (2018). A practical guide to using qualitative research with randomized controlled trials.

[13] Owen JG, Hankins JD, Iams V, Berghella JS, Sheffield A, Perez-Delboy R.S, Egerman DA, Wing M, Tomlinson R, Silver SM, Ramin ER, Guzman M, Gordon HY, How EJ, Knudtson J M, Szychowski S, Cliver and J. C. Hauth (2009). "Multicenter randomized trial of cerclage for preterm birth prevention in high-risk women with shortened midtrimester cervical length." Am J Obstet Gynecol 201(4): 375 e371-375 e378. 10.1016/j.ajog.2009.08.015 PMC2768604 19788970

[14] RCOG (2011). The investigation and treatment of couples with recurrent Firsttrimester and second-trimester miscarriage Greentop guideline 17.

[15] Saccone G, Rust O, Althuisius S, Roman A, Berghella V (2015). Cerclage for short cervix in twin pregnancies: systematic review and meta-analysis of randomized trials using individual patient-level data. Acta Obstet Gynecol Scand.

[16] Sedgwick P (2014). Bias in observational study designs: prospective cohort studies. BMJ.

[17] van’t Hooft J, Duffy JM, Daly M, Williamson PR, Meher S, Thom E (2016). A Core outcome set for evaluation of interventions to prevent preterm birth. Obstet Gynecol.

[18] Ventolini G, Neiger R (2008). Management of painless mid-trimester cervical dilatation: prophylactic vs emergency placement of cervical cerclage. J Obstet Gynaecol.

[19] Ehsanipoor RM, Seligman NS, Saccone G, Szymanski LM,Wissinger C, Werner EF, Berghella V. Physical Examination-Indicated Cerclage: A Systematic Review and Meta-analysis. Obstet Gynecol 2015;126(1):125-35.10.1097/AOG.0000000000000850. 10.1097/AOG.0000000000000850 26241265

